# Research progress on the mechanisms of *Panax* *ginseng* and its active components in maintaining skin homeostasis and disease intervention

**DOI:** 10.1186/s13020-026-01407-y

**Published:** 2026-05-07

**Authors:** Lu Ding, Zihan Wang, Nanqi Hou, Yuchi Zhou, Hongyu Qi, Zirui Li, Xueyan Li, Jiaojiao Xue, Siyu Song, Zeyu Wang, Daqing Zhao, Rui Jiang, Xiangyan Li

**Affiliations:** 1 Research Center of Traditional Chinese Medicine, The First Affiliated Hospital of Changchun University of Chinese Medicine, 1478 Gongnong Street, Changchun, 130021 Jilin People’s Republic of China; 2 Northeast Asia Research Institute of Traditional Chinese Medicine, Changchun University of Chinese Medicine, 1035 Boshuo Road, Changchun, 130117 Jilin China

**Keywords:** *Panaxginseng*, Indirect pharmacology, Active metabolites, Gut microbiota, Exosomes, Skin delivery systems

## Abstract

*Panax* *ginseng* (*Panax* *ginseng* C.A. Meyer) is a classic herbal medicine widely utilized in dermatological management, yet its precise therapeutic mechanisms have only recently begun to be fully elucidated. This review systematically synthesizes the pharmacological roles of ginseng’s active components, including ginsenosides, polysaccharides, and gintonin, in maintaining skin homeostasis and treating various cutaneous pathologies. Emerging evidence suggests that the efficacy of ginseng extends beyond direct molecular interactions, exhibiting the distinct characteristics of "indirect pharmacology." Specifically, parent ginsenosides function primarily as natural prodrugs that require microbial biotransformation into high-affinity active metabolites, such as Compound K and Rh2, to exert potent therapeutic effects. Functionally, these components regulate skin health by mobilizing endogenous host defense systems, particularly through the activation of the Nrf2/ARE antioxidant pathway and the suppression of inflammatory cascades via the NF-κB, MAPK, and IL-1 signaling networks. Furthermore, they remodel the systemic microenvironment through the gut-skin axis, facilitating metabolic homeostasis to improve skin barrier function. Notably, biotransformation rates and ultimate therapeutic efficacy are highly dependent on individualized variables, such as host age, dietary patterns, and baseline disease states. This systemic regulation has demonstrated robust efficacy in intervening in complex pathologies, including atopic dermatitis, psoriasis, and skin cancer. Additionally, this article addresses the translational bottleneck of low bioavailability and evaluates recent advances in novel skin delivery systems, specifically those utilizing ginseng-derived exosomes. Collectively, this review provides a scientific framework for understanding the systemic actions of ginseng, supporting its development as a precision botanical therapy for dermatological applications.

## Introduction

**Fig. 1:**
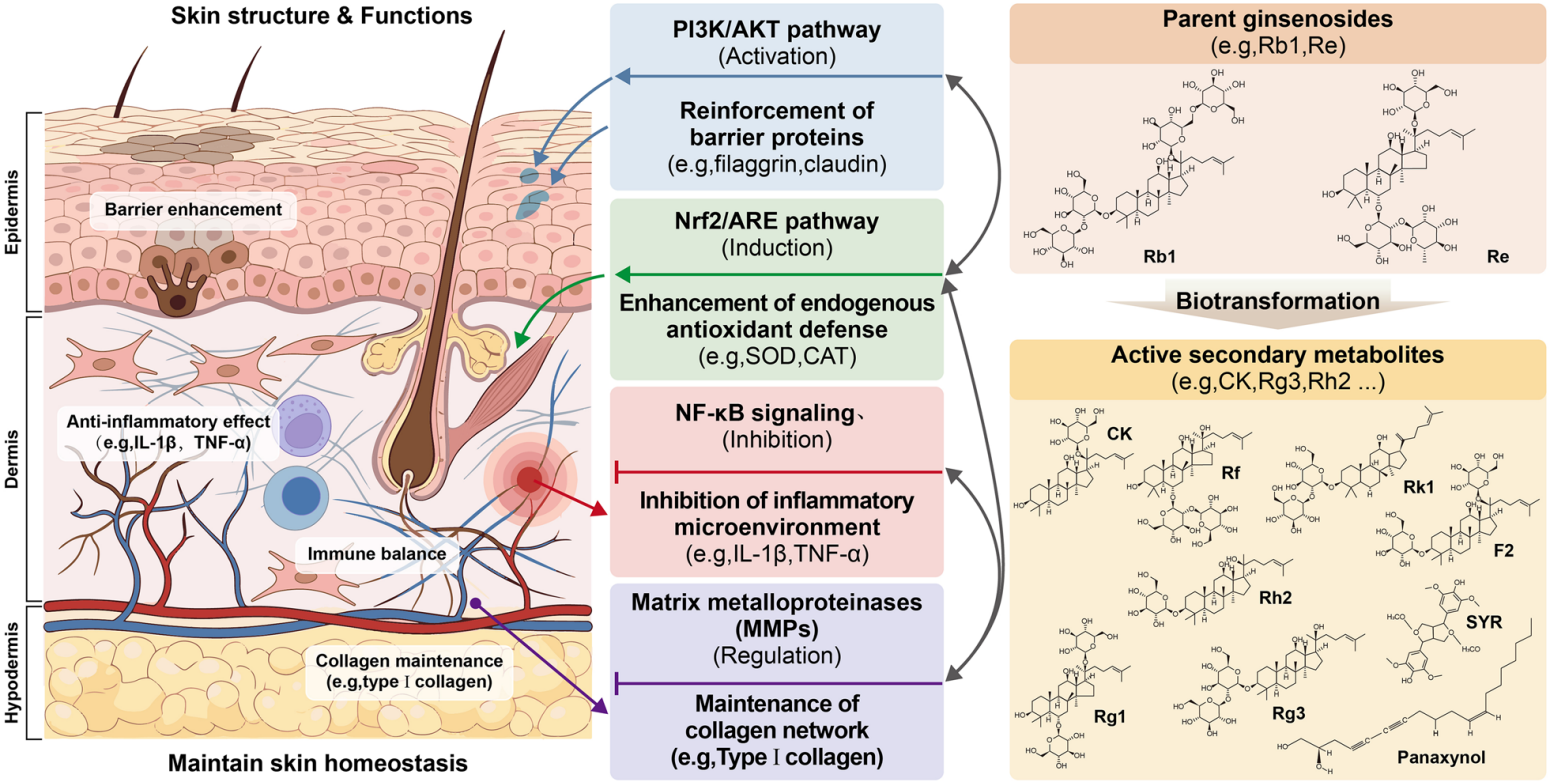
A unified network delineating the basic skin structure, chemical constituents of Panax ginseng, and their targeted homeostatic regulatory mechanisms. This schematic integrates the biological correspondences between structural features of the skin and the active substances of ginseng. The left panel illustrates the three core layers of the skin (epidermis, dermis, and subcutaneous tissue) alongside their fundamental functions in maintaining hydration (TEWL), thermoregulation, and immune equilibrium. The right panel systematically categorizes the parent ginsenosides (e.g., Rb1, Re) and their highly active secondary metabolites (e.g., CK, Rg3, Rh2). The central network visually maps how these specific constituents maintain cutaneous homeostasis via multi-target mechanisms. These include fortifying barrier proteins via PI3K/Akt activation, enhancing endogenous antioxidant defenses through the Nrf2/ARE pathway, suppressing the inflammatory microenvironment by modulating NF-κB, and preserving the collagen network via MMP regulation

The skin, the human body's largest organ, serves as the primary physical and immunological barrier against environmental challenges [1, 2]. This complex organ prevents transepidermal water loss and pathogen infiltration through a resilient "brick-and-mortar" arrangement of corneocytes and intercellular lipids within the stratum corneum, complemented by a "molecular sieve" formed by tight junction proteins in the granular layer [3–5]. It also actively contributes to immune surveillance, sensory transmission, and thermoregulation [6–8]. The maintenance of skin homeostasis depends on tightly regulated and evolutionarily conserved signaling pathways, such as NF-κB and MAPK, which enable resident immune cells to monitor microenvironmental changes, distinguish harmless stimuli from potential threats, and sustain local immune equilibrium [9]. As illustrated in Fig. 1, skin health emerges from the coordinated interaction between its multilayered structure and complex functional networks.

However, the modern living environment poses severe challenges to this delicate equilibrium [10–14]. The intertwining of air pollutants, ultraviolet (UV) radiation, and global population aging aggravates the risk of skin barrier damage, chronic inflammatory skin diseases, and photoaging [15]. Despite the established efficacy of current chemical drugs and biologics, their long-term application is often associated with limitations such as drug resistance, skin irritation, and systemic immunosuppression. Consequently, there is an urgent need to seek natural therapeutic strategies characterized by milder action and multidimensional interventional capabilities [16, 17].

In this context, the traditional medicinal plant Ginseng exhibits substantial potential [18–20]. Its long history of use and extensive pharmacological studies have demonstrated broad biological activities across cardiovascular, neurological, and oncological domains [21–23]. Recent investigations have increasingly focused on the material basis underlying its pharmacological effects, encompassing various active constituents such as ginsenosides, gintonin, polysaccharides, volatile oils, and peptides [24–26]. These components show distinctive value in topical skin applications, acting not through single targets but via synergistic mechanisms that enhance skin barrier function, counter oxidative stress, modulate inflammatory responses, and delay cellular senescence, thereby providing a strong scientific foundation for their use in skin health [26–30]. However, as research has matured, it has been increasingly recognized that the protective effects of ginseng on the skin are not achieved solely through the direct target binding of parent molecules, but rather involve complex "indirect pharmacology" mechanisms. Specifically, many high-molecular-weight or hydrophilic ginseng components function as natural prodrugs, requiring biotransformation by the gut or skin microbiota in vivo to generate secondary metabolites with superior bioavailability and potency. These "active intermediate substances", such as Compound K and other rare ginsenosides, frequently serve as the primary effector molecules responsible for the observed therapeutic outcomes [31]. Furthermore, the action of ginseng is characterized by significant "indirect regulation," meaning it maintains skin homeostasis "indirectly" by inducing endogenous antioxidant enzyme systems (e.g., the Nrf2 pathway) or regulating systemic networks such as the "gut-skin axis," rather than merely directly killing pathogens or repairing cells [32, 33].

Despite growing evidence supporting the dermatological applications of ginseng, most existing reviews remain limited to describing its direct effects. These works often lack a systematic integration of the "indirect pharmacology" perspective and fail to fully elucidate the pivotal roles that intermediate metabolites and indirect regulatory networks play in countering increasingly severe environmental challenges to the skin [10–14]. Therefore, this review aims to establish a comprehensive framework encompassing both direct and indirect actions. We focus on dissecting how the active components of ginseng generate key intermediate substances through biotransformation and exert indirect protective effects by inducing endogenous defense systems and modulating systemic axes (such as the gut-skin axis). This article systematically elucidates these mechanisms within the context of intervening in pathologies such as atopic dermatitis, psoriasis, and skin cancer. Furthermore, we explore novel delivery systems designed to target these indirect mechanisms, providing a clear roadmap for future research and industrial development in this field.

## Main active components of ginseng and skin permeability

### Ginsenosides

Ginsenosides are the principal pharmacologically active constituents of ginseng [34]. According to their aglycone structures, they are categorized into dammarane-type, oleanolic acid-type, and ocotillol-type saponins [35]. Dammarane-type saponins are further divided into protopanaxadiol (PPD, e.g., Rb1, Rc, Rd, Rg3, Rh2) and protopanaxatriol (PPT, e.g., Re, Rf, Rg1, Rg2) subtypes, each exhibiting distinct biological properties due to structural variations [31]. However, when scrutinized from the perspective of "indirect pharmacology," the diverse biological effects exhibited by these ginsenosides due to structural differences are often not mediated solely by their parent forms. Research by Yang et al. profoundly revealed that naturally occurring "major ginsenosides" (e.g., Rb1, Re) typically possess low membrane permeability and actually function as "prodrugs" in vivo. They require biotransformation by the gut microbiota-stripping off sugar moieties-to generate "active intermediate substances" with higher bioavailability and potency, thereby exerting their therapeutic effects. This "indirect action" mediated by metabolic intermediates is particularly evident in skin protection: taking PPD-type saponins as an example, they typically exhibit strong antioxidant and anti-inflammatory potential [36], but their mechanism is not simply direct free radical scavenging, but rather the mobilization of the host's endogenous defense systems. For instance, Ding et al. found that the key active component ginsenoside Rg3 can "indirectly" enhance the activities of endogenous antioxidant enzymes such as superoxide dismutase (SOD) and glutathione peroxidase (GSH-Px), thereby effectively alleviating UVB-induced skin damage [37]. Conversely, PPT-type saponins excel in promoting extracellular matrix metabolism, delaying cellular senescence, and fostering collagen synthesis, effects that similarly rely on the remodeling of complex cellular signaling networks [38]. Studies by Kim et al. demonstrated that ginsenosides Rg1, Re, and Rb1 have all been confirmed to synergistically promote collagen synthesis and inhibit its degradation by regulating fibroblast nuclear receptors (e.g., PPAR-δ) and associated signaling pathways (e.g., ERK/AP-1) [39–42]. This epigenetic regulation of gene expression further confirms that ginsenosides maintain skin elasticity and structural integrity primarily through "indirect pharmacological" pathways.

To clarify the indirect pharmacology of ginsenosides as prodrugs, it is essential to delineate the biological agents and kinetics driving their conversion into active intermediates. Following systemic (oral) administration, the gut microbiota serves as an indispensable driver of this biotransformation. Specific intestinal bacterial strains, notably Bacteroides and Bifidobacterium, secrete highly active β-glucosidases and β-galactosidases. Through sequential deglycosylation, these enzymes efficiently cleave macromolecular parent ginsenosides (e.g., Rb1 and Re) into highly potent secondary metabolites, such as Compound K (CK) [43, 44]. In contrast, upon topical application, skin-resident commensals, such as *Staphylococcus epidermidis*, and endogenous epidermal glycosidases exhibit a measurable degree of biotransformation capacity. However, their cumulative catalytic efficiency and enzymatic conversion rates remain substantially lower than those observed within the gastrointestinal microbiome [45]. Furthermore, across both intestinal and cutaneous environments, the specific composition of the local microbiota dictates the kinetics of metabolite activation. A highly diverse microbiome enriched with glycosidase-producing strains exponentially accelerates the deglycosylation of parent ginsenosides. In contrast, under conditions of dysbiosis, the subsequent decline in key enzymatic activities drastically decelerates this bioconversion. This ecological impairment directly restricts the yield of active intermediates, thereby compromising the ultimate pharmacological efficacy of the botanical intervention [46].

### Gintonin

In addition to ginsenosides that require biotransformation, Gintonin, a unique glycolipoprotein complex identified in ginseng, represents another mode of "indirect pharmacology" in skin protection-namely, exerting effects by mobilizing the body's pre-existing endogenous signaling networks [47–49]. Unlike saponins that act through metabolites, Gintonin functions primarily as an exogenous ligand for G protein-coupled lysophosphatidic acid (LPA) receptors (e.g., LPA1, LPA3), mimicking the function of endogenous growth factors [50]. It triggers a rapid intracellular calcium signaling cascade ([Ca^2^⁺]i transients), which subsequently activates downstream kinases such as ERK and Akt, thereby "awakening" the self-repair capabilities of skin cells [51, 52]. In skin biology, this regulation of host endogenous signal systems translates into pronounced reparative and matrix-remodeling effects: it effectively drives the proliferation and migration of keratinocytes and fibroblasts, accelerating wound healing; simultaneously, it stimulates the synthesis of endogenous collagen and hyaluronic acid, maintaining structural integrity [53, 54]. Furthermore, in terms of inflammation regulation, studies indicate that Gintonin improves the inflammatory microenvironment of atopic dermatitis by modulating autotaxin activity, highlighting its indirect role in regulating immune homeostasis [55]. Of significant translational value, studies by Lee et al. successfully isolated Gintonin from red ginseng marc (an industrial by-product) and confirmed its in vitro potential against UV-induced cellular senescence and in promoting wound healing [56, 57]. In summary, Gintonin is not a simple nutritional supplement but constitutes a skin protection network complementary to ginsenosides by specifically activating the host "calcium signaling-matrix synthesis" axis mediated by LPA receptors.

### Ginseng polysaccharides

Ginseng polysaccharides is another essential class of bioactive macromolecules in ginseng, recognized for their potent antioxidant and immunomodulatory activities [58]. Given their high molecular weight, which limits direct transdermal absorption, modern research has gradually revealed that their skin protective effects rely primarily on "indirect pharmacology" mechanisms-specifically, maintaining skin homeostasis through systemic immune remodeling and "gut-skin axis" regulation. Evidence indicates that ginseng polysaccharides promote longevity in model organisms by modulating conserved signaling pathways such as TOR, offering new molecular insight into systemic aging delay [27, 59]. Crucially, research by Huo and colleagues provided definitive evidence for this "indirect action": they confirmed that specific colon-targeted ginseng polysaccharide microspheres significantly remodeled the gut microbiota structure after release. This systemic suppression of inflammatory cytokines via the "gut-skin" metabolic axis represents a microbiome-based distal regulatory mechanism that is central to the anti-inflammatory and anti-aging effects of ginseng polysaccharides [60]. At the local skin level, the action of polysaccharides also involves indirect regulation of the microenvironment. Studies by Chen and Zhao et al. indicate that ginseng polysaccharides (especially oligosaccharide fractions) not only scavenge free radicals, but, more importantly, act as "prebiotics" to regulate the skin surface microbiota balance [61]. By activating endogenous barrier repair signals, they promote keratinocyte differentiation and ceramide synthesis, thereby effectively reducing transepidermal water loss (TEWL) and repairing damaged barriers [62]. This dual mechanism-"gut microbiota remodeling" internally and "skin microecological regulation" externally-fully embodies the unique value of ginseng polysaccharides as "biological response modifiers" in skin health.

### Other components

In addition to saponins, polysaccharides, and Gintonin, minor components in ginseng-such as volatile oils, peptides, and amino acids-play non-negligible synergistic roles within the "indirect pharmacology" network, despite their lower content. Recent multi-omics studies have revealed that the mechanisms of these components often involve systemic metabolic reprogramming and microbiome modulation [63–65]. Ginseng volatile oil, a key lipid-soluble active fraction, not only possesses direct antibacterial properties but, more critically, enhances the organism's resilience through "indirect" pathways [66–68]. Wang and Qiao reported that it significantly extends healthspan in model organisms by activating the cellular "autophagy" program-the cell's self-cleaning and repair mechanism-and upregulating endogenous antioxidant enzymes such as SOD. This mode of action, characterized by enhancing host endogenous defenses rather than mere exogenous supplementation, constitutes the fundamental basis for its efficacy in combating skin oxidative stress [67]. On the other hand, ginseng-derived bioactive peptides exhibit superior cell penetration and metabolic regulatory capabilities. Zhu et al. demonstrated that ginseng oligopeptides (GOPs) act as "mitochondrial function regulators." By activating the NAD +/SIRT1/PGC-1α signaling axis, they improve fibroblast senescence from the source of energy metabolism [69]. These minor components, in concert with major bioactive substances, form a multidimensional protective net of "antimicrobial homeostasis-metabolic regulation-endogenous defense," significantly enhancing the skin's adaptability and self-repair capacity under complex environmental stress.

After systematically elucidating the "biotransformation" of ginsenosides, the "endogenous signal simulation" of Gintonin, the "immune-gut-skin axis regulation" of polysaccharides, and the synergistic mechanisms of other components, we can clearly define the essence of ginseng in maintaining skin health: it is not the linear action of a single component, but a complex pharmacological network dependent on "intermediate metabolic substances" and "indirect systemic regulation." To present this system more concisely and systematically, Table 1 summarizes the core active components across dimensions such as component classification, key intermediate substances, primary dermatological effects, and indirect molecular mechanisms, aiming to provide a clear theoretical map for the subsequent in-depth discussion of their specific applications in skin diseases.

**Table 1:** Indirect pharmacological properties of ginseng active components and their metabolic intermediates in skin homeostasis regulation

Category	Key active components (representative substances)	Primary dermatological effects (based on indirect mechanisms)	Key molecular mechanisms & targets	References
Active intermediate substances (Biotransformation Products)	Compound K (CK) (Terminal metabolite of Rb1)	Anti-photoaging/Psoriasis Intervention (Structural remodeling & Immune blockade)	Cell cycle reprogramming: Downregulates p21/p53 and upregulates p63 to reverse the cellular senescence program Immune source blockade: Inhibits IL-23 secretion from dendritic cells, severing the IL-23/Th17 axis Receptor Activation:Activates Glucocorticoid Receptor (GR) to exert steroid-like anti-inflammatory effects	[162, 191, 249]
	Ginsenoside F2 (Rare secondary saponin)	Atopic Dermatitis/Barrier Repair (Gut-Skin Axis Systemic Regulation)	Gut-skin axis remodeling: Modulates gut microbiota composition and enriches short-chain fatty acid (SCFA)-producing bacteria to indirectly suppress cutaneous inflammation Effector phase interception: Inhibits IL-17 production by γδ T cells	[194, 250]
	Ginsenoside Rg3 (PPD metabolite)	Anti-Skin Cancer/AD Pruritus Relief (Epigenetic & Th2 Regulation)	Epigenetic modification: Specifically downregulates HDAC3 and increases c-Jun acetylation to inhibit tumor Epithelial-Mesenchymal Transition (EMT) Immune polarization regulation: Blocks Th2 cell polarization and reduces IL-4/IL-13 release at the source	[174, 205]
	Ginsenoside Rh2 (Rare saponin)	Anti-Skin Cancer/Broad-spectrum Anti-inflammatory (Stem Cell Targeting & Autophagy)	Targeting tumor roots: Inhibits the Wnt/β-catenin pathway and eliminates Lgr5 + Cancer Stem Cells (CSCs) Mobilizing endogenous defense: Induces cytoprotective autophagy	[140, 208]
Parent/Precursor Ginsenosides (Major existing forms)	Ginsenoside Rb1/Re	Barrier Construction/Matrix Homeostasis (Initiating Endogenous Synthesis)	Initiating endogenous synthesis: Activates PI3K/Akt and PPAR-δ pathways to upregulate Claudin-1 and Occludin expression Awakening differentiation programs: Re activates Caspase-14, promoting the degradation of Filaggrin into Natural Moisturizing Factors (NMF)	[96, 251]
	Ginsenoside Rg1	Anti-Oxidative Stress (Enzyme System Mobilization)	Endogenous enzyme mobilization: Activates the Keap1-Nrf2-ARE axis to induce host expression of SOD, CAT, and GSH-Px	[33, 101]
Non-saponin components	Gintonin (Glycolipoprotein Complex)	Wound Healing/Anti-Aging (Receptor-mediated Regeneration)	Endogenous receptor mimicry: Acts as an exogenous ligand for LPA receptors (LPA1/3) to trigger intracellular [Ca^2^⁺]i transients 2. Growth signal mimicry: Activates ERK/Akt pathways to drive cell migration and matrix regeneration	[24, 53]
	Ginseng Polysaccharides (GPs)	Systemic Anti-inflammatory/Barrier Maintenance (Microecology & Metabolic Regulation)	Microbiome modulation: Acts as a prebiotic to remodel gut/skin microbiota and systemically reduce inflammatory load Barrier signal activation: Promotes ceramide synthesis to enhance physical defense	[60, 105]
	Panaxynol (Polyacetylenes)	Anti-photoaging/Anti-inflammatory (ROS Source Blockade)	ROS source blockade: Directly inhibits NADPH oxidase 4 (NOX4) activity, cutting off the source of ROS generation	[127, 131]
	Syringaresinol (SYR) (Lignans)	Anti-Natural Aging (Cellular Quality Control)	Autophagy flux remodeling: Upregulates LC3B to restore protein clearance capabilities in senescent cells	[231]

### Regulatory mechanisms of ginseng active components on skin health

The maintenance of skin homeostasis is a highly intricate physiological process involving the orderly proliferation and differentiation of epidermal keratinocytes, the integrity of physical and molecular barriers, the balance of intracellular redox status, and the precise regulation of inflammatory responses [70–75]. Disruption of this network by external stressors such as UV radiation or pollutants, or by internal factors such as aging, can lead to various pathological skin conditions [76–78]. A profound analysis of this process reveals that the protective effects of ginseng exhibit distinct characteristics of "indirect pharmacology" (Fig. 1) As illustrated in Fig. 1, this mechanism is not a linear action of single molecules but rather a multidimensional network dependent on "biotransformation" and "systemic regulation".

### Skin barrier repair effects

The structural integrity of the skin barrier forms the fundamental basis for maintaining body homeostasis and protecting against environmental challenges [79, 80]. This complex defense system comprises two primary layers: the outer stratum corneum, which establishes a stable “brick-and-mortar” architecture through terminally differentiated corneocytes and intercellular lipids such as ceramides, cholesterol, and fatty acids, effectively minimizing transepidermal water loss and preventing penetration of external substances [81–84]; and the underlying granular layer, which acts as a selective “molecular sieve” formed by tight junction proteins including Claudin-1, Occludin, and ZO-1, regulating paracellular ion and small molecule transport to preserve epidermal osmotic balance [85, 86]. Disruption of this barrier directly increases TEWL and susceptibility to external irritants, leading to or aggravating skin dryness, hypersensitivity, and various inflammatory dermatoses [87, 88].

In the investigation of barrier repair mechanisms mediated by ginseng active components, emerging evidence highlights the pivotal role of "indirect pharmacology": rather than functioning merely as direct physical fillers, ginseng relies heavily on the generation of "active intermediate substances" and the mobilization of the host's "endogenous repair systems"[89, 90]. Pivotal to exerting barrier repair efficacy are the intermediate metabolites generated via biotransformation. Recent studies by Tan and Kim et al. indicate that biotransformed products of ginsenosides (particularly ginseng berry saponins), as well as metabolite Compound K-enriched fractions (e.g., BIOGF1K), exhibit efficacy in enhancing epidermal barrier function that is far superior to their parent forms [91, 92]. This indicates that parent ginsenosides function as "prodrugs" that must be transformed into these highly active intermediate substances to efficiently initiate the barrier repair program. Beyond the generation of metabolites, ginsenosides mobilize endogenous repair mechanisms by activating intracellular signaling networks. Among them, ginsenoside Rb1 has been the most systematically studied and is regarded as an upstream regulator of the PI3K/Akt signaling pathway [93–95]. By activating this conserved endogenous pathway, Rb1 indirectly achieves dual repair: promoting the proliferation of dermal papilla cells to support appendage renewal [96, 97], and inducing intranuclear gene transcription to upregulate tight junction proteins (Claudin-1, Occludin) [98, 99]. Parallel to this signaling cascade, the action of Ginsenoside Re is characterized by the "awakening" of the keratinocyte terminal differentiation program. Research by Oh et al. revealed that Re specifically upregulates Filaggrin and activates Caspase-14 enzymatic activity, thereby mobilizing the cell's own Natural Moisturizing Factor (NMF) generation mechanism to optimize water retention functions from within [100]. Furthermore, in combating UVB-induced damage, multiple ginsenosides indirectly reverse barrier dysfunction by synergistically inducing the expression of Filaggrin, Loricrin, and Aquaporin 3 (AQP3) [101–103]. Expanding the repair network beyond saponins, ginseng polysaccharides and Gintonin demonstrate strategies based on "microenvironment regulation." Research by Sun and Gao et al. reveals that ginseng polysaccharides can improve metabolic homeostasis by modulating the "intermediary system" of the gut and skin microbiota, thereby indirectly supporting skin barrier maintenance [59, 104, 105]. In contrast, Gintonin functions as an exogenous ligand for endogenous LPA receptors, mimicking growth signaling to promote cell migration and matrix synthesis [106]. Taken together, ginseng achieves deep repair of the skin barrier through a multidimensional indirect pharmacological network characterized by "intermediate metabolite transformation, endogenous signal awakening, and microecological regulation."

### Antioxidantive stress effects

Oxidative stress represents a central pathophysiological mechanism underlying skin photoaging, chronological aging, and inflammatory dermatoses. Its results from an imbalance between the production and clearance of intracellular reactive oxygen species (ROS) [107–109]. Under physiological conditions, ROS act as signaling molecules in cellular regulation; however, excessive ROS accumulation caused by exogenous stimuli such as UV radiation and environmental pollutants, or endogenous factors such as mitochondrial dysfunction, induces lipid peroxidation, protein carbonylation, and DNA damage. The resulting accumulation of malondialdehyde (MDA), a terminal product of lipid peroxidation, serves as a critical indicator of compromised cell membrane integrity [110–114]. This molecular damage ultimately manifests as reduced skin elasticity, wrinkle formation, and impaired barrier function [115–118]. Unlike the direct free radical scavenging mode of traditional antioxidants (e.g., Vitamin C), ginseng active components primarily exert their effects through an "indirect pharmacology" mechanism-namely, by "awakening" or "mobilizing" the host's powerful endogenous antioxidant enzyme systems, rather than relying solely on chemical neutralization by exogenous molecules. Ginsenoside Rg1 serves as the quintessential example of this "indirect antioxidant" action [119, 120]. Gao et al. detailed that Rg1 functions not as a direct free radical scavenger, but as an "upstream activator" of the Keap1-Nrf2-ARE endogenous defense pathway [33, 121]. Mechanistically, Rg1 induces a conformational change in Keap1, facilitating the dissociation and subsequent nuclear translocation of the transcription factor Nrf2. Upon entering the nucleus, Nrf2 binds to the Antioxidant Response Element (ARE) to initiate the transcription of a downstream array of "cytoprotective proteins," including SOD, CAT, and GSH-Px [122–124]. Li and colleagues confirmed in a UVB-induced injury model that while Rg1 treatment did not directly participate in chemical scavenging, it indirectly reduced intracellular ROS levels by 40–50% and inhibited MDA production by upregulating the activities of these endogenous enzymes [101, 125]. This indirect mechanism, dependent on host gene expression, often exhibits more durable cytoprotective efficacy compared to direct antioxidants. On the other hand, Panaxynol represents a distinct indirect strategy: "source blockade"[126, 127]. NADPH oxidase 4 (NOX4) serves as the critical "generator" of ROS bursts in the skin [128–130]. A recent review by Li and Wang indicates that Panaxynol specifically inhibits the enzymatic activity of NOX4, thereby intercepting ROS generation at the most upstream level, rather than passively scavenging them downstream [131]. This mechanism forms a perfect complement to the "scavenging enhancement" strategy of Rg1. Further research by Liu et al. confirmed that by inhibiting the metabolic source NOX4 and coordinating with the upregulation of endogenous SOD activity, Panaxynol effectively reconstructs the redox equilibrium of damaged skin [132, 133].

Collectively, ginseng active components construct a multidimensional antioxidant defense network that is independent of exogenous consumption, utilizing the dual indirect mechanisms of "Nrf2-mediated endogenous enzyme induction" and "NOX4-mediated source inhibition." This mode of combating oxidative stress by regulating the cell's intrinsic metabolic programs represents the core embodiment of ginseng's "indirect pharmacology" in skin protection.

### Anti-inflammatory effects

Skin inflammation is a pathological process driven by the activation of immune cascades in response to external stimuli [134, 135], where NF-κB acts as a central regulator whose excessive activation drives the release of factors such as TNF-α and IL-6, as well as matrix degradation [136–138]. In the investigation of ginseng's anti-inflammatory mechanisms, the emerging perspective of "indirect pharmacology" has revealed a critical principle: the potent anti-inflammatory efficacy of ginseng is often not dependent on parent ginsenosides, but is rather dominated by their biotransformed "active intermediate substances." These intermediate substances possess superior cell membrane permeability and targeting capabilities, enabling them to penetrate deep into cells or achieve precise anti-inflammatory effects through systemic microenvironmental regulation.

First, rare ginsenoside Rh2, functioning as a prototypical "active intermediate substance," demonstrates a broad-spectrum capacity to remodel the inflammatory microenvironment [139]. Studies by Pan and Peng et al. reveal that Rh2 does not merely block a single receptor but operates through a dual "indirect braking" mechanism: on one hand, it prevents IκBα degradation, thereby indirectly sequestering NF-κB from nuclear translocation; on the other hand, it inhibits TAK1 phosphorylation to blockade upstream p38 MAPK signaling transduction [140, 141]. This multi-target inhibitory strategy has been confirmed in psoriasis and atopic dermatitis models to systematically reduce IL-6 and TNF-α levels, exhibiting efficacy far superior to that of untransformed parent ginsenosides [142, 143]. Secondly, Ginsenoside Rf embodies a dual indirect regulatory strategy of "anti-inflammation combined with endogenous repair" [144, 145]. Recent research by Kang and colleagues revealed that within the inflammatory microenvironment, Rf not only indirectly reduces IL-1β and IL-6 secretion by suppressing the NF-κB pathway, but more critically, it "awakens" the self-repair program of keratinocytes [28]. Studies confirmed that Rf specifically upregulates the expression of FLG and AQP3 while inhibiting matrix MMP-1 [28]. This mode of reconstructing physical barriers by mobilizing the cell's own protein synthesis while simultaneously inhibiting inflammatory destruction represents a unique manifestation of ginseng's "indirect pharmacology" in maintaining skin structural integrity. Addressing inflammation associated with photoaging, metabolites Rk1 and Rk3 achieve precise intervention through a "structure–function" coupling mechanism [133, 146]. Liu and Wan et al. confirmed that Rk1 specifically binds to the PI3K catalytic domain, severing the PI3K/AKT/NF-κB cascade from the upstream, thereby reducing UVB-induced inflammatory cytokines and MMP-2/9 expression by over 50% [133, 147]. Meanwhile, Rk3 complements Rk1 by regulating MMP-1/3 expression, indirectly protecting the collagen network from inflammatory degradation [146, 148–150]. This dual regulation of inflammatory signaling and matrix metabolism further confirms the central role of intermediate substances in maintaining skin homeostasis. Beyond these pathways, the modulation of the interleukin-1 (IL-1) network constitutes another critical dimension of ginseng's indirect anti-inflammatory properties. The IL-1 signaling axis functions as a primary initiator driving chronic epidermal inflammation and aberrant keratinocyte hyperproliferation. Accumulating evidence indicates that active ginseng metabolites, particularly Compound K (CK) and Rg1, exert a dual-pronged intervention on the IL-1 network via both "production blockade" and "signal interception."

Mechanistically, specific ginsenosides substantially attenuate the maturation and secretion of IL-1βat its source by impeding the assembly of the NLRP3 inflammasome. Furthermore, these metabolites demonstrate the capacity to intercept downstream signal transduction by specifically inhibiting Interleukin-1 receptor-associated kinase 1 (IRAK-1), a pivotal kinase within the IL-1 receptor (IL-1R) complex. By disrupting this axis, ginseng active components effectively prevent the nuclear translocation of pro-inflammatory transcription factors, such as NF-κB, thereby mitigating the inflammatory cascade in diseased skin tissues [151–153]. Concurrently, at the level of receptor signal transduction, the terminal metabolite Compound K has been demonstrated to specifically suppress the activation of interleukin-1 receptor-associated kinase-1 (IRAK-1). Given that IRAK-1 is an indispensable kinase downstream of the IL-1 receptor (IL-1R), its targeted inhibition fundamentally uncouples the transmission of IL-1 signaling to the downstream NF-κB cascade [154, 155]. Such precise molecular blockade provides a compelling scientific rationale for positioning ginseng as a promising candidate in corticosteroid-free anti-inflammatory therapeutics.

In summary, the anti-inflammatory action of ginseng is a systemic process driven by high-potency intermediate substances. Whether through the broad-spectrum signaling blockade mediated by Rh2, the mobilization of endogenous barrier repair by Rf, the targeted intervention against photoaging-associated inflammation by Rk1/Rk3, or the upstream interception of the initiating IL-1 network by CK and Rg1, these findings collectively demonstrate a consistent pharmacological paradigm. Ginseng must undergo biotransformation or precisely modulate host systems to achieve efficient indirect regulation within complex inflammatory networks.

### Anti-photoaging effects

Skin photoaging is a complex pathological condition resulting from cumulative damage caused by prolonged UV exposure. UVA, with its strong dermal penetration, induces excessive ROS generation, disrupts mitochondrial function, and directly damages DNA, thereby activating multiple cellular stress signaling pathways [156–158]. This sustained damage promotes the cascade release of inflammatory mediators and markedly upregulates matrix metalloproteinases (MMPs), including MMP-1, MMP-3, and MMP-9, leading to extensive degradation of collagen and elastic fibers and compromising epidermal barrier integrity. Clinically, these processes manifest as skin roughness, deep wrinkles, loss of elasticity, and diminished repair capacity [159–161]. In the research on ginseng's role in combating this aging process, emerging evidence once again points to the core of "indirect pharmacology": namely, that the anti-photoaging efficacy of ginseng is primarily driven by its biotransformed "active intermediate substances" These metabolites achieve the reversal of skin aging by remodeling cell cycle programs and mobilizing mitochondrial defense systems.

As a primary effector, the protopanaxadiol-type metabolite CK demonstrates the capability of "indirect reprogramming" of cellular senescence programs [162, 163]. Recent research by Zhang, Li et al. elucidated the core mechanism of CK: it functions not merely as a simple antioxidant, but precisely regulates the pivotal "senescence brake" signaling axis of p53-p21. By downregulating p21 expression, CK alleviates UVA-induced cell cycle arrest while simultaneously upregulating p63 to maintain the proliferative potential of keratinocytes [162]. Crucially, this study unveils a strategy of "combinatorial indirect pharmacology": when CK, functioning as an "intermediate substance," is combined with retinol derivatives (HPR/VAPA), the two generate synergistic efficacy. While significantly inhibiting MMP-1/3 expression, the formulation leverages CK's anti-inflammatory properties to counteract the irritating side effects of retinol. This metabolite-based synergy provides novel strategies for photoaging treatment [28, 162]. Acting on a distinct mitochondrial front, the rare metabolite Ginsenoside Rh1 exerts anti-aging effects by "mobilizing" the mitochondrial defense system. Recent work by Su and Wang et al. revealed that the target of Rh1 resides in the mitochondrial Sirt3 protein (a deacetylase). Acting as an upstream agonist, Rh1 activates Sirt3, promoting the nuclear translocation of the transcription factor Nrf2 and inducing the expression of downstream HO-1. This mechanism essentially represents the mobilization of the host cell's endogenous "mitochondrial-antioxidant" dual defense axis. Experiments confirmed that silencing the Sirt3 gene directly abolishes the protective effects of Rh1, providing robust evidence that this component must rely on the response of host endogenous proteins to exert its anti-photoaging effects [164].

In conclusion, the mechanism by which ginseng active components combat photoaging is essentially the result of the coordinated division of labor among multiple "highly active intermediate substances." With CK regulating the cell cycle and matrix metabolism, and Rh1 activating the mitochondrial Sirt3 defense axis, these mechanisms jointly construct a multi-level anti-aging network.

This section systematically elucidates the molecular network through which ginseng active components maintain skin homeostasis via multi-target synergy. To provide a global perspective, Table 2 summarizes the key pathways, functional effects, and experimental evidence across four core physiological processes: barrier repair, antioxidant activity, anti-inflammation, and anti-photoaging/aging. This table clearly reveals that ginseng exerts its potent pleiotropy not through a single pathway, but through an indirect pharmacological closed loop of "prodrug transformation-intermediate substance generation-endogenous system mobilization." This establishes a solid physiological and pharmacological foundation for its intervention potential in the various skin pathological states to be discussed in the subsequent section.

**Table 2:** Summary of the regulatory effects of ginseng active components on key physiological processes for skin health

Core physiological process	Active ingredient	Action pathway	Regulatory direction	Functional effect	Research model	References
Barrier repair	Ginsenoside Rb1	PI3K/Akt → Claudin-1/Occludin	Activation	Enhances tight junction integrity and increases trans-epithelial electrical resistance	Keratinocyte monolayer model	[93–95, 98]
	Ginsenoside Re	Caspase-14/Filaggrin	Activate/Upregulate	Promote stratum corneum maturation and increase natural moisturizing factor production	HaCaT keratinocytes	[100]
	Synergistic action of multiple saponins	Filaggrin/Involucrin/AQP3	Upregulation	Jointly repair UVB-induced barrier damage, reduce TEWL, and restore ceramide levels	BALB/c hairless mice, HaCaT cells	[100, 103]
Antioxidant	Ginsenoside Rg1	Keap1/Nrf2/ARE	Activation	Induces expression of antioxidant enzymes such as SOD, CAT, and GSH-Px, reducing intracellular ROS	HaCaT cells, UVB-damaged model	[33, 121–124]
	Ginsenoside (Volatile Oil)	NOX4/ROS	Inhibition	Suppresses ROS production at the source, reducing MDA levels in skin tissue	UVB-induced mouse photodamage model	[128–131]
Anti-inflammatory	Ginsenoside Rh2	NF-κB & p38 MAPK	Inhibition	Broad-spectrum inhibition of pro-inflammatory factors such as IL-6 and TNF-α expression and secretion	LPS/UV-induced inflammatory model, macrophages	[139–143, 252–254]
	Ginsenoside Rf	NF-κB & ECM Metabolism	Inhibition/Promotion	Exhibits both anti-inflammatory and reparative functions, downregulates IL-1β/IL-6 while promoting collagen synthesis	TNF-α/IFN-γ-stimulated NHDFs and NHEKs	[28, 144, 145]
	Ginsenoside Rk1	PI3K/Akt/NF-κB	Inhibition	Specific inhibition of this pathway significantly reduces IL-1β, TNF-α, and MMPs	UVB-induced mouse photoaging model	[133, 147–150]
Anti-photoaging/anti-aging	Ginsenoside CK	p53/p21 & MMPs	Downregulation/Inhibition	Delays cellular aging, inhibits collagen degradation, synergizes with retinol	UVA-induced fibroblast senescence model	[162, 163]
	Ginsenoside Rh1	Sirt3/Nrf2/HO-1	Activation	Enhances antioxidant defense via the mitochondrial pathway to alleviate photoaging	UV-induced skin photoaging model	[164]

### Research progress of ginseng active components in skin diseases

**Fig. 2:**
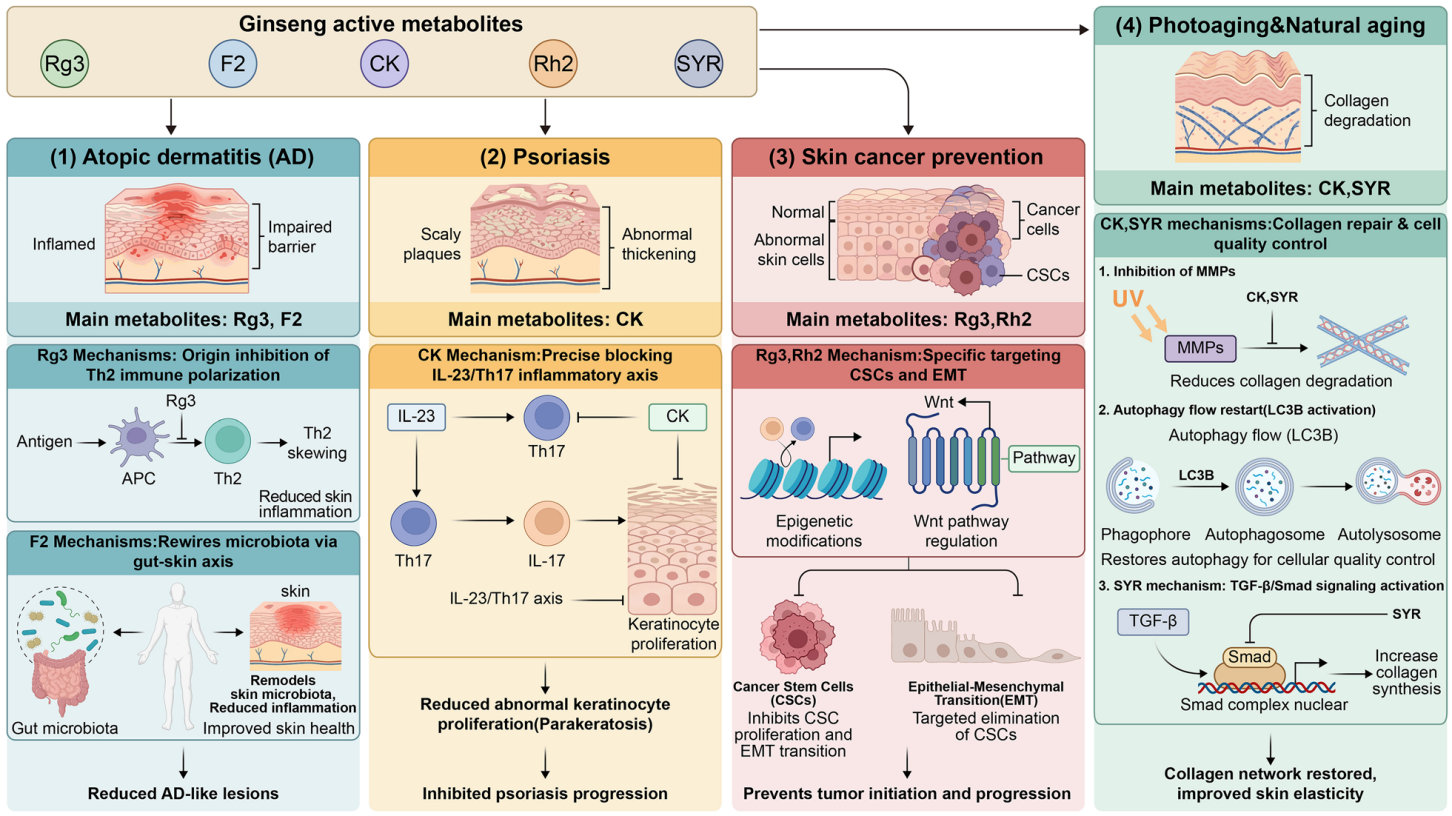
Molecular intervention landscape of active ginseng metabolites against specific dermatological diseases. This diagram establishes direct mechanistic correlations between specific ginseng metabolites and distinct pathological states. (**1**) In atopic dermatitis, the intermediate Rg3 intercepts Th2 immune polarization at its source, while F2 remodels the microecology via the gut-skin axis; (**2**) In psoriasis, CK precisely blockades the IL-23/Th17 inflammatory axis, thereby suppressing aberrant keratinocyte hyperproliferation; (**3**) In skin cancer chemoprevention, Rg3 and Rh2 specifically target cancer stem cells and inhibit tumor epithelial-mesenchymal transition (EMT) through epigenetic modifications and Wnt pathway modulation; (**4**) In photoaging and chronological aging, metabolites such as CK and SYR orchestrate collagen repair and cellular quality control by inhibiting matrix metalloproteinases (MMPs), restoring autophagy flux (LC3B), and activating TGF-β/Smad signaling

In summary, the preceding sections systematically reveal that ginseng functions not merely as a simple skin "nutrient," but as a homeostatic regulator based on "indirect pharmacology." Its core mechanism relies on "active intermediate substances" generated via biotransformation. By "mobilizing" host endogenous defense systems and "remodeling" systemic microenvironments, it achieves multidimensional interventions ranging from barrier repair (Sect.  3.1), redox resetting (Sect.  3.2), and precise inflammatory regulation (Sect.  3.3) to the reversal of photoaging (Sect.  3.4). This closed-loop mechanism of "prodrug transformation-intermediate substance generation—endogenous system response" provides a unique pharmacological perspective for understanding its therapeutic efficacy in complex pathological states. Building on this foundation, this section will provide an in-depth review of the therapeutic interventions of ginseng active components in common dermatological disorders, including atopic dermatitis, psoriasis, and skin cancer. We will focus on exploring how these highly active metabolites combat disease progression through systemic indirect regulation (Fig. 2).

To provide a concise overview of the evidence for ginseng's "indirect pharmacology" in disease treatment, Table 3 systematically summarizes core findings across models of atopic dermatitis, psoriasis, skin cancer, and photoaging. This table clearly delineates the specific components playing pivotal roles, the research models employed, and the underlying key indirect molecular mechanisms, offering a comprehensive evidentiary framework for the subsequent detailed discussion of specific diseases.

**Table 3:** Preclinical evidence of ginseng active components in skin diseases

Disease model	Active ingredient	Research model	Primary effect	Core mechanism	References
AD	Ginsenoside Rg3	DNFB/Ovalbumin-Induced Mouse Model	Reduces severity of skin lesions, decreases scratching, lowers serum IgE levels	Inhibits Th2 cell polarization, reduces IL-4, IL-13, CCL17, CCL22	[172–175]
	Ginsenoside F2	DNCB-induced mouse model	Improves skin barrier function and reduces inflammation	Reshapes gut/skin microbiota, increasing short-chain fatty acid-producing bacteria	[32, 179–184]
	Gintonin	DNFB-induced mouse model	Alleviates dermatitis symptoms and inhibits mast cell infiltration	Suppresses autophagy enzyme activity and modulates LPA receptors	[176–178]
Psoriasis	Ginsenoside CK	IMQ-induced mouse model	Reduces scaling, erythema, and skin thickening	Inhibits the IL-23/Th17 axis and suppresses keratinocyte hyperproliferation	[191–193]
	Ginsenoside Rg1	IMQ-induced mouse model	Reduce skin inflammation and hyperplasia	Antagonize the NF-κB pathway, downregulate TNF-α and IL-6	[196]
	Ginsenoside F2	TPA-induced mouse skin inflammation model	Suppresses skin inflammation and neutrophil infiltration	Inhibits γδ T cell production of IL-17, reducing neutrophil ROS	[194, 196]
Skin cancer	Ginsenoside Rg3	CSCC Cell Lines and Models	Inhibition of Tumor Cell Migration and Invasion	Downregulation of HDAC3 Increases c-Jun Acetylation and Suppresses EMT	[205, 255]
	Ginsenoside Rh2	Squamous Cell Carcinoma Stem Cell Model	Inhibits cancer stem cell self-renewal and survival	Suppresses the Wnt/β-catenin pathway, downregulates Lgr5-positive stem cells	[206, 207, 256]
	Ginsenoside Re	Melanoma cell lines and mouse models	Inhibition of melanoma growth and melanin synthesis	Downregulation of MITF and its downstream target genes (TYR, TRP-1, TRP-2)	[209–211]
Photoaging	Ginsenoside CK	UVA-Induced Fibroblast Senescence Model	Synergizes with Retinol Derivatives to Promote Anti-Apoptosis and Collagen Synthesis	Downregulates p53/p21, Upregulates p63, Inhibits MMPs, and Scavenges ROS	[162, 216]
	Ginsenoside Rk1	UVB-induced HaCaT/HDF cells and hairless mouse models	Alleviate oxidative stress and inflammation, reduce collagen degradation	Inhibit PI3K/AKT/NF-κB pathways, decrease MMP-1/2/9	[133, 217–219]
	Ginsenoside C-Y	UVB-Induced Hairless Mouse Model	Reduces wrinkles, enhances elasticity, and inhibits pigmentation	Activates TGF-β/Smad and Nrf2/ARE pathways while suppressing MMP-1 and melanin production	[220]
Natural aging	SYR	H₂O₂-Induced Senescence Model in HaCaT Cells	Delays cellular senescence and reduces collagen degradation	Upscales autophagy marker protein LC3B and inhibits MMP-2/MMP-9 activity	[24]
	PAM	UVA-Induced HFF-1 Cell Model	Protects against DNA oxidative damage and delays cellular senescence	Activates the Nrf2 pathway, enhancing SOD and CAT activity	[221, 228]

### Atopic dermatitis

AD is a chronic inflammatory skin disorder characterized by severe pruritus, xerosis, and eczematous lesions. Currently, the pathogenesis of AD is widely recognized as being bidirectionally driven by an "outside-in" epidermal barrier defect and an "inside-out" immune dysregulation, which collectively precipitate a vicious cycle exacerbated by cutaneous microbiome dysbiosis [165–168]. The former denotes that aberrations in barrier proteins, notably filaggrin (FLG), predispose the skin to external irritants, whereas the latter describes how a Th2-skewed immune deviation (marked by the release of IL-4 and IL-13) compromises the epidermal barrier from within. The therapeutic potential of ginseng and its active metabolites lies in their capacity to concurrently intercept both pathogenic trajectories via an "indirect pharmacological" mechanism. Specifically, targeting the "outside-in" pathway, parent ginsenosides (such as Rb1 and Re) induce the endogenous synthesis of FLG and ceramides, thereby fortifying the physical defense of the stratum corneum [89]. Conversely, addressing the "inside-out" pathway, highly active intermediate metabolites generated post-bioconversion (e.g., Rg3 and Rh2) fundamentally suppress Th2 cell polarization and downregulate the expression of thymic stromal lymphopoietin (TSLP), thereby mitigating internal inflammatory erosion [169, 170]。 This strategy-eschewing reliance on a singular molecular target in favor of utilizing biotransformed, highly active intermediate metabolites to systematically remodel both the immune microenvironment and the gut-skin axis-constitutes the fundamental basis for ginseng's efficacy in managing AD [32, 171, 172].

In the regulation of the core Type 2 immune response, the metabolic intermediate Rg3 demonstrates precise "indirect inhibition" of Th2 cells. Studies indicate that Rg3 blocks Th2 cell polarization and activation at the source, significantly reducing IL-4 and IL-13 release. Consequently, it downregulates the expression of Th2 chemokines (CCL17, CCL22) in keratinocytes, thereby indirectly interrupting the itch-scratch cycle [172–175]. Working in concert with these immunomodulators, Rh1 and Gintonin exert broad-spectrum effects by blocking the MAPK/NF-κB signaling pathway, inhibiting the infiltration of eosinophils and mast cells, and suppressing IgE release [55, 176–178]. Perhaps the most compelling evidence for "indirect pharmacology" is provided by Ginsenoside F2, which acts distally via the "gut-skin axis."Research by Li and Dong et al. confirmed that oral F2 remodels the dysbiotic gut microbiota structure. Rather than acting directly on the skin, it enriches beneficial bacteria that produce short-chain fatty acids (SCFAs, especially propionate), improving systemic metabolic status. Upon entering circulation, these metabolites "indirectly" enhance skin barrier function and suppress inflammation [32, 179]. Complementing these major pathways, small molecule components like p-coumaric acid target the inhibition of the TSLP/NF-κB axis, while the metabolite Compound K-enriched fraction (BIOGF1K) synergistically inhibits mast cell degranulation [180–184].

Although the evidence derived from animal models and in vitro experiments has preliminarily constructed a mechanistic landscape of ginseng active components (especially metabolic intermediates) in the multi-pathway synergistic treatment of AD, the current chain of evidence still lacks high-quality clinical validation. Therefore, future research should focus on verifying the translational efficiency of these "indirect mechanisms" in humans to advance the clinical application of ginseng preparations.

### Psoriasis

Psoriasis is a chronic inflammatory skin disorder characterized by immune dysregulation. Its key pathological features include keratinocyte hyperproliferation, aberrant differentiation, and infiltration of inflammatory cells dominated by neutrophils, macrophages, and T cells [185–187]. The IL-23/IL-17 inflammatory axis serves as a central driver in both the initiation and persistence of the disease [188–190]. Addressing this intricate immune network, the therapeutic advantage of ginseng lies in its strategic "indirect pharmacology": specifically, it relies on "active intermediate substances" generated via biotransformation to precisely "sever" and remodel critical nodes within the immune cascade.

Ginsenoside CK demonstrates a capability for "source blockade" against the IL-23/IL-17 axis. Research by Lee and Zhang et al. confirmed that CK does not act through nonspecific anti-inflammation, but precisely targets the immune initiation stage: it inhibits dendritic cells from secreting IL-23, thereby indirectly blocking the downstream activation of Th17 cells and the release of IL-17. This metabolite-mediated source control effectively inhibited abnormal keratinocyte proliferation and significantly improved erythema and scaling in animal models, embodying the typical mechanism of "prodrug transformation-metabolite effect" [191–193]. During the effector phase, rare saponins play a crucial role of "indirect interception." Park et al. discovered that the transformed product Ginsenoside F2 directly targets the core producers of IL-17-γδ T cells-inhibiting their IL-17 production and reducing neutrophil ROS generation, thus blocking the inflammatory storm from the effector end [194]. Meanwhile, recent research by Zheng et al. indicates that the rare saponin fraction from ginseng berry (GFRS, rich in biotransformed secondary saponins) broadly inhibits the secretion of IL-6, TNF-α, and IL-17A. This further confirms that transformed rare components possess stronger immune remodeling activity than their parent mixtures [195]. Providing synergistic regulation, Ginsenoside Rg1 antagonizes the NF-κB signaling pathway. Shi et al. confirmed that Rg1 primarily reduces the inflammatory burden of the tissue microenvironment indirectly by downregulating TNF-α and IL-6 [196].

Currently, clinical perspectives increasingly conceptualize psoriasis as a systemic inflammatory disease inextricably linked to multiple comorbidities—notably metabolic syndrome and cardiovascular diseases—which frequently share overlapping systemic inflammatory pathways with cutaneous lesions. The systemic regulatory attributes of ginseng harbor substantial translational potential for intervening in these psoriatic comorbidities. Substantial meta-analyses and preclinical studies confirm that active ginseng components (such as Rb1, Rg1, and their downstream metabolites) not only ameliorate local psoriatic lesions but also remodel the "gut-liver-skin axis" by modulating lipid metabolism, preserving vascular endothelial integrity, and attenuating systemic oxidative stress [32, 197]. Consequently, this multi-target intervention indirectly downregulates systemic inflammatory markers (e.g., TNF-α, CRP) and mitigates cardiovascular complication risks, highlighting the therapeutic rationale for incorporating ginseng into the comprehensive management of psoriasis and its systemic comorbidities [198]. Ultimately, this paradigm shift—moving from the isolated treatment of localized skin lesions toward the holistic remodeling of systemic inflammation, perfectly encapsulates the multi-target advantages of ginseng in the comprehensive management of psoriasis.

### Skin cancer

Skin cancer is among the most prevalent malignancies worldwide, with its development closely associated with UV radiation–induced DNA damage, genomic instability, and aberrant activation of oncogenic signaling pathways [199–203]. In the mechanistic investigation of ginseng against skin cancer, the perspective of "indirect pharmacology" offers novel insights: ginseng's anti-tumor effects do not rely on broad-spectrum cytotoxicity (akin to chemotherapeutic agents), but are primarily achieved by biotransformed "highly active intermediate substances." These metabolites execute precise intervention by remodeling the epigenetic landscape, targeting cancer stem cells, and reprogramming transcriptional networks [204].

In the context of Cutaneous Squamous Cell Carcinoma (CSCC), the metabolic intermediate Rg3 exhibits unique "epigenetic remodeling" capabilities. Zhang and his team confirmed that Rg3 does not directly kill cells but acts as an "epigenetic regulator" to specifically downregulate Histone Deacetylase 3 (HDAC3). This modulation results in the indirect elevation of acetylation levels of the transcription factor c-Jun, thereby effectively inhibiting the Epithelial-Mesenchymal Transition (EMT) process of CSCC cells at the gene transcription level, blocking tumor invasion and metastasis at the source [205]. Targeting the root of tumor recurrence, the rare ginsenoside Rh2 precisely affects Cancer Stem Cells (CSCs) through a "dual indirect mechanism." Liu et al. discovered that Rh2 can "mobilize" the intracellular autophagy program, an endogenous defense mechanism, while synergistically inhibiting the Wnt/β-catenin signaling pathway. This dual strike significantly weakens the self-renewal capacity of Lgr5-enriched CSCs [206–208]. Unlike traditional therapies, Rh2 offers the potential to fundamentally suppress skin cancer recurrence by eliminating these "seed cells" that are often in a dormant or drug-resistant state. In highly malignant melanoma, Ginsenoside Re exemplifies the "transcriptional reprogramming" of key oncogenic drivers. Hwang and Bang et al. pointed out that Re does not merely inhibit the activity of melanogenic enzymes but directly targets their upstream master regulator-Microphthalmia-associated Transcription Factor (MITF). By downregulating MITF and its downstream target gene network (Tyrosinase, TRP-1/2), Re simultaneously curbs the proliferative drive and pigment synthesis capacity of melanoma at the transcriptional level, achieving a "two birds with one stone" indirect blocking effect [204, 209–211]. Notably, while the interventional mechanisms of active ginseng components have been well-established in squamous cell carcinoma and melanoma, investigations into their therapeutic application for basal cell carcinoma (BCC) remain conspicuously sparse. The oncogenesis of BCC is heavily reliant on the aberrant activation of the Hedgehog (Hh) signaling cascade, frequently driven by deleterious mutations in the PTCH1 or SMO genes [212]. Given that specific ginsenosides (e.g., Rh2 and Rg3) are proven modulators of the Wnt/β-catenin axis, a critical developmental pathway that exhibits extensive crosstalk with Hh signaling and is intricately linked to cancer stem cell maintenance—it presents a compelling frontier for future research to elucidate whether these highly active metabolites can function as natural Hh pathway antagonists [206, 213]. Furthermore, synergizing these compounds with advanced transdermal delivery systems to formulate topical adjuvants for superficial BCC represents a crucial trajectory for expanding the dermatological oncology repertoire of ginseng. Ultimately, through rigorous mechanistic exploration targeting pivotal networks such as the Hh cascade, ginseng and its active biotransformed derivatives hold profound promise as novel candidate strategies for the chemoprevention and adjuvant management of cutaneous malignancies.

### Photoaging

Skin photoaging is an exogenous aging process resulting from long-term and repeated exposure to ultraviolet radiation (primarily UVB and UVA). Clinically, it manifests as wrinkle formation, skin roughness, loss of elasticity, and pigmentation disorders. The core molecular mechanisms involve UV-induced ROS overproduction, abnormal activation of the MMPs leading to extracellular matrix (especially collagen) degradation, and failure of the endogenous antioxidant defense system [214, 215]. Addressing this multifactorial process, ginseng's anti-photoaging strategy relies heavily on "indirect pharmacology": specifically, utilizing biologically transformed "highly active intermediate substances" to simultaneously initiate the dual programs of "structural repair" and "endogenous antioxidant defense."

As the final metabolite of protopanaxadiol-type saponins, Compound K (CK) exemplifies the precise regulation of collagen metabolic homeostasis. Zhang and Kim et al. confirmed that rather than merely supplementing collagen, CK inhibits the upstream expression of matrix metalloproteinase-1 (MMP-1) and inflammatory mediators (COX-2) while simultaneously activating the fibroblast's intrinsic synthetic machinery to promote Type I collagen neogenesis. This metabolic regulation mode of balancing synthesis and degradation effectively reverses the core manifestations of photoaging [162, 216]. Expanding the defensive scope, rare ginsenosides such as Rk1 and C-Y (a metabolite of Rb2) achieve systemic photoprotection by mobilizing endogenous signaling pathways. Liu et al. discovered that Rk1 specifically modulates the PI3K/AKT/NF-κB axis, blocking UVB-induced oxidative stress and inflammatory storms at the level of signal transduction, thereby restoring collagen synthesis [133, 217–219]. Distinguished by a dual-action mechanism, the metabolite C-Y not only scavenges ROS but also synergistically activates TGF-β1/Smad (the master pathway for collagen synthesis) and Nrf2/ARE (the master pathway for antioxidant defense). This coordinated activation enhances the cell's intrinsic antioxidant defense while repairing the dermal structure, and further exerts anti-melanogenic effects to achieve multidimensional photoprotection [220]. Complementing the saponin fraction, non-saponin components further fortify this defense network [28]. Ginseng polysaccharides reinforce the physical skin barrier by regulating the release of inflammatory factors, while ginseng phenolic acids specifically activate the Nrf2 pathway, mobilizing intranuclear DNA repair mechanisms to significantly alleviate UVA-induced oxidative damage and delay cellular senescence [221].

In conclusion, the anti-photoaging efficacy of ginseng is not a mere summation of single components, but is founded on the "synergistic mobilization" of endogenous pathways-such as TGF-β/Smad (structural) and Nrf2 (defense)-by "active intermediate substances." By "remodeling" the cell's own metabolic and repair programs, these metabolites construct a robust anti-photoaging defense system.

### Skin aging

Distinct from photoaging driven by environmental factors, endogenous aging (natural aging) is a complex physiological degeneration process driven by genetic programs, characterized by telomere shortening, proteostasis imbalance, and declined autophagic function, clinically manifesting as delayed barrier repair and structural atrophy [222–225]. Addressing this endogenous degeneration, ginseng's intervention strategy embodies typical "systemic indirect pharmacology"-namely, by "awakening" the dormant defense and quality control mechanisms within cells to retard the aging process.

Targeting the chronic low-grade inflammation and oxidative accumulation associated with aging, ginseng active components implement a dual indirect clearance strategy. Recent research by Kang et al. confirmed that Ginsenoside Rf not only scavenges ROS but significantly inhibits the formation of the Senescence-Associated Secretory Phenotype (SASP) [28, 226, 227]. By downregulating IL-1β and IL-6, it severs the inflammatory encroachment of senescent cells upon the surrounding microenvironment. Parallel to this modulation, the Panax Acid Mixture (PAM) activates Nrf2, the pivotal antioxidant hub, to induce the expression of SOD and CAT. This mode of mobilizing cellular enzyme systems to combat oxidative stress proves more durable than exogenous antioxidants [221, 228]. In the context of extracellular matrix homeostasis, the metabolic intermediate F2 plays a pivotal protective role. Hwang and Lee et al. found that rare ginsenoside F2 specifically inhibits constitutive MMP-1 expression, thereby protecting collagen from age-dependent degradation. Simultaneously, ginseng extract restarts the Type I collagen synthesis program in fibroblasts by activating the Smad signaling pathway, thereby improving skin density [229, 230]. Beyond matrix protection, the ginseng-derived non-saponin component Syringaresinol (SYR) restores cellular quality control capabilities by remodeling autophagy flux. Choi and Kim et al. reported that SYR significantly upregulates the expression of the autophagy marker protein LC3B. As autophagy is the endogenous mechanism for clearing damaged proteins and organelles, SYR activates this mechanism to indirectly reduce MMP-2/9 activity and delay cellular senescence, representing an essential cytological basis for ginseng's action against endogenous aging [231]. Expanding the scope of resource utilization, Gintonin, extracted from red ginseng marc by Lee et al., functions as an exogenous ligand for LPA receptors. By mimicking endogenous growth signals to activate the LPA receptor axis, it re-drives cell proliferation and suppresses aging phenotypes within the senescent environment. This strategy of turning waste into treasure with a well-defined mechanism offers a novel direction for the development of anti-aging ginseng products [24].

In summary, ginseng active components construct a multidimensional anti-aging defense network by targeting core biological processes such as antioxidant defense (Nrf2), maintenance of matrix homeostasis (Smad/MMP), and activation of autophagy (LC3B). Specifically, the inhibition of SASP by Rf, the remodeling of autophagy by SYR, and the activation of endogenous receptors by Gintonin fully demonstrate that the essence of ginseng's anti-aging action lies in "mobilizing host endogenous systems." These findings not only deepen the understanding of pharmacological mechanisms but also provide a unified theoretical explanation for the interventions in various skin diseases summarized in Table 3**.** Future translational research should be dedicated to validating the human efficacy of these "indirect mechanisms" in 3D skin models and clinical trials.

### Impact of host factors on the absorption and efficacy of active ginseng components

Although the multi-target interventional mechanisms of ginseng metabolites have been systematically elucidated above, their actual in vivo absorption rates and ultimate pharmacological efficacies remain highly contingent upon the individualized physiological states of the host. Given that the core tenet of "indirect pharmacology" hinges on biotransformation, variables such as age, dietary patterns, and baseline disease states emerge as critical modulators of ginseng's therapeutic potency.

Primarily, advancing age is frequently accompanied by a progressive decline in the diversity of both intestinal and cutaneous microbiomes, coupled with a deterioration in endogenous enzymatic activities. This ecological senescence directly decelerates the conversion rate of parent ginsenosides into active intermediate entities (e.g., CK and Rh2). Consequently, older demographics may exhibit compromised bioavailability and diminished clinical benefits compared to their younger counterparts under identical dosing regimens [232, 233]. Furthermore, dietary patterns exert a profound remodeling effect on systemic absorption. A high-fiber diet functions as a natural prebiotic, selectively fostering the proliferation of beneficial β-glucosidase-producing microbiota, such as Bifidobacterium. Such diet-driven microbial optimization can significantly expedite the degradation and activation of precursor saponins, thereby amplifying their systemic anti-inflammatory and antioxidant efficacies [234, 235]. Ultimately, baseline disease states fundamentally alter the pharmacokinetics of drug delivery and bioconversion. For instance, during the acute exacerbation phases of AD or psoriasis, the epidermal barrier function is severely compromised. While this "leaky" barrier may paradoxically augment the passive transdermal permeation of macromolecular parent ginsenosides, these inflammatory dermatoses are invariably accompanied by severe local cutaneous dysbiosis-typified by the over-colonization of Staphylococcus aureus and a precipitous drop in microbial diversity [236]. The disruption of this microecological niche severely attenuates the local biotransformation capacity of the skin, rendering the permeated compounds unable to be efficiently converted into bona fide, highly active effector molecules [237]. Therefore, the future clinical development of ginseng-based botanical therapies must imperatively integrate these host variables. Strategies such as prior in vitro bioconversion or co-administration with microbiome-modulating agents are essential to actualize precision, individualized treatments. To provide a more intuitive delineation of how these factors orchestrate indirect pharmacological processes, Table 4 systematically summarizes the specific impacts of administration routes and diverse host variables on the biotransformation efficiency and ultimate clinical efficacy of ginsenosides.

**Table 4:** Impact of host and administration route variables on the biotransformation and ultimate efficacy of ginsenosides

Core variable category	Specific variable state	Alterations in microecological and enzymatic environments	Impact on the bioconversion and absorption of precursor ginsenosides	Impact on ultimate clinical efficacy	References
Administration route & converting agent	Systemic administration (Oral)	Dominated by gut microbiota (e.g., Bacteroides, Bifidobacterium), exhibiting exceptionally high β-glucosidase activity	Rapid bioconversion kinetics; efficiently cleaves macromolecular parent ginsenosides into highly active secondary metabolites (e.g., CK)	High systemic bioavailability, resulting in robust systemic anti-inflammatory and immunomodulatory efficacy	[43]
	Topical administration (Transdermal)	Mediated by skin-resident commensals and endogenous epidermal glycosidases; overall catalytic efficiency and enzymatic capacity are strictly limited	Local bioconversion rates are significantly restricted, yielding low quantities of active intermediate metabolites from parent ginsenosides	Relies on parent compounds to exert limited effects; the direct application of pre-converted, highly active rare ginsenosides in topical delivery systems is strongly recommended	[45]
Host physiological variables	Advancing age (Senescence)	Progressive decline in the diversity of intestinal and cutaneous microbiomes, coupled with a deterioration of endogenous enzymatic activities	Decelerated bioconversion kinetics, leading to a diminished yield of secondary metabolites	Under identical dosing regimens, older demographics generally exhibit compromised bioavailability and diminished clinical benefits compared to younger cohorts	[232]
	High-fiber diet	Functions as a prebiotic to remodel the gut microbiota, selectively fostering the proliferation of beneficial β-glucosidase-producing strains	Substantially accelerates the degradation and activation of precursor saponins, elevating the plasma concentration of active intermediates	Amplifies systemic anti-inflammatory and antioxidant efficacies, thereby enhancing overall therapeutic outcomes	[234]
Baseline disease states	Acute inflammatory dermatoses (e.g., AD, Psoriasis)	Epidermal barrier "leakiness"; accompanied by severe local cutaneous dysbiosis (e.g., over-colonization of Staphylococcus aureus and a precipitous drop in microbial diversity)	Impaired barrier augments passive transdermal permeation; however, dysbiosis severely attenuates local biotransformation capacity	Permeated compounds fail to be efficiently converted into potent effector molecules at the lesion site, restricting overall anti-inflammatory efficacy	[237]

### Research on skin delivery systems for ginseng active components

Although ginseng active components (particularly highly active metabolic intermediates) exhibit immense pharmacological potential in the field of skin health, the practical realization of their "indirect pharmacology" efficacy is constrained by a core bottleneck: achieving effective transdermal delivery and targeted accumulation across the skin barrier. Specifically, this challenge presents a dichotomy: On one hand, parent saponins (e.g., Rb1) are characterized by high molecular weight (> 800 Da) and high hydrophilicity, hindering their penetration through the lipid matrix of the stratum corneum (SC). On the other hand, while the more biologically potent "intermediate substances" (e.g., Compound K) possess increased lipophilicity, their extremely poor aqueous solubility severely limits their stability in formulations and subsequent bioavailability [238].

To overcome these physicochemical limitations and significantly enhance delivery efficiency, researchers have developed various advanced carrier systems (Table 5). Studies by Wang et al. indicate that ethosome-based gel formulations of protopanaxatriol saponins (PTS) achieved an encapsulation efficiency of 93%, significantly inhibiting melanogenesis in UVB-induced tanning models [239]. Similarly, research by Jin and Liu et al. pointed out that encapsulating total ginsenosides in liposomes and niosomes not only achieved high encapsulation rates but also significantly enhanced the transmembrane flux of the drugs. This highly efficient delivery strategy not only protects active components from photothermal degradation but also functionally manifests as a significant inhibition of photoaging markers (such as lipid peroxidation), thereby ensuring the effective activation of "endogenous defense"[238]. Regarding the optimization of delivery kinetics, Zou and his team developed a novel nanoparticle system based on ginsenoside self-assembly. This system demonstrated astonishing cellular uptake efficiency, achieving rapid intracellular penetration within 5 min and maintaining sustained release for up to 48 h. This kinetic profile of "rapid onset-sustained maintenance" perfectly matches the requirement in "indirect pharmacology" for continuous intervention on metabolic regulatory targets (such as Nrf2 or NF-κB) [240]. In terms of carrier stability and biocompatibility, the red ginseng nanoemulsion and gel systems optimized by Pfleger et al. exhibited excellent biocompatibility while maintaining physicochemical stability for up to 12 weeks, ensuring that active components are gently released and act upon skin cells in their most active state [241]. In the aspect of structural repair, the collagen transdermal patch designed by Wang et al. embodies the concept of "carrier-drug synergy": experimental data showed that the optimized patch formulation exhibited superior skin permeability and retention rates. This highly efficient local delivery enabled Rb1 to work synergistically with the collagen carrier, significantly inhibiting α-MSH-induced melanogenesis, achieving a comprehensive upgrade from "physical delivery" to "biological efficacy"[242].

**Table 5:** Primary skin delivery systems for ginseng active ingredients and their application effects

Delivery system type	Active ingredient	Core advantages/mechanisms	Efficiency metrics	References
Ethosome gel	PTS	Optimized PTS formulation and evaluated its whitening efficacy in a UVB-induced tanning mouse model	PTS ethosome gel effectively inhibits UVB-induced melanin production and exhibits a high encapsulation rate	[239]
GSL and GSN	GS	Enhancing transdermal absorption of GS via liposomes and liposome-like carriers, and evaluating its anti-photoaging effects	GSL-7 significantly inhibits UV-induced skin lipid peroxidation and reduces expression of inflammatory cytokines and MMPs	[238]
Nanoemulsions and gels	KRG	Optimizing KRG's multiphase and monophase delivery systems while evaluating their antioxidant capacity and biocompatibility	The KRG delivery system maintained stability over 12 weeks and demonstrated excellent biocompatibility with skin cells	[241]
Nanoparticles	Ginsenosides and Insulin	Development of nanoparticles formed by ginsenoside self-assembly for transdermal insulin delivery	Nanoparticles can temporarily permeate cells within 5 min and sustain insulin release for 48 h, maintaining stable blood glucose levels	[240]
Collagen transdermal patch	Ginsenoside Rb1	Develop a collagen transdermal patch for topical delivery of Rb1 and evaluate its skin-whitening efficacy	Rb1 significantly inhibits α-MSH-stimulated melanin production in B16 cells; the optimized patch formulation demonstrates excellent permeability and retention rate	[242]

Table 5 systematically summarizes mainstream delivery strategies such as liposomes, nanoemulsions, hydrogels, and microneedles. The intervention of these technological means has fundamentally resolved the dilemma of ginseng active components being "unable to enter or stay" from a physicochemical perspective. By drastically improving penetration efficiency and targeted retention, these technologies provide solid technical support for achieving precise indirect regulation within the complex skin physiological environment. In recent years, exosomes have emerged as a promising class of natural nanocarriers, forging novel technological avenues for the transdermal delivery of bioactive ginseng compounds. Current investigative efforts are largely bifurcated: while certain studies have endeavored to encapsulate ginsenosides within mammalian-derived exosomes to augment tissue targeting and bioavailability, a surging paradigm shift has spotlighted the direct isolation of "ginseng-derived exosome-like nanoparticles" (GrDENs) from the botanical tissue itself. Architecturally characterized by a lipid bilayer, GrDENs are intrinsically enriched with a sophisticated payload of ginsenosides (e.g., Rg1, Rb1, and Re), specialized lipids, and functional RNAs. When benchmarked against conventional synthetic liposomes, these natural nanovesicles exhibit markedly superior transdermal penetrability and cellular internalization efficiencies [243, 244]. Recent elucidations by Choi et al. demonstrate that GrDENs can robustly traverse the stratum corneum barrier and undergo rapid internalization by epidermal keratinocytes. Once intracellular, they effectively reverse UV-induced photoaging and inflammatory cascades by downregulating the activator protein-1 (AP-1) signaling axis and curtailing reactive oxygen species (ROS) generation [245, 246]. This dual-identity delivery paradigm-functioning concomitantly as both a "natural carrier" and an "active therapeutic agent"-provides a compelling biomimetic blueprint for the future development of topical ginseng formulations [247].

## Conclusions and future prospects

This review, through a systematic examination of ginseng active components, culminates in Fig. 1 and 2, which constructs a panoramic molecular landscape ranging from fundamental health maintenance to complex disease intervention. Retrospectively reviewing this precise regulatory network, we posit that the pharmacological essence of ginseng is not the linear action of a single component, but a synergistic regulatory network jointly orchestrated by multi-components including ginsenosides, polysaccharides, Gintonin, and volatile oils. As illustrated by the representative components in Fig. 1, this system demonstrates potent homeostatic maintenance capabilities through distinct functional modules: the barrier construction module represented by Rb1 and Re reinforces physical defenses; the redox mobilization module represented by Rg1 and Panaxynol mobilizes endogenous enzymes; the inflammation inhibition module represented by Rh2 and Rf remodels the immune microenvironment; and the anti-aging module represented by CK and SYR controls cell cycle quality. These quintessential examples constitute the solid foundation of ginseng in maintaining skin health and intervening in diseases.

This holistic mode of action, based on multi-targets and multi-pathways, offers a novel perspective for understanding ginseng's efficacy in complex pathological states such as atopic dermatitis, psoriasis, and skin cancer. Distinct from the modern medical paradigm of pursuing potent single-target blockade, ginseng exhibits a unique characteristic of "systemic indirect pharmacology," extending far beyond simple receptor binding. At the material level, parent saponins act as natural prodrugs that must be transformed into highly active intermediate substances (e.g., CK, F2) to achieve precise efficacy; at the spatial level, components like F2 achieve remote regulation from the gut to the skin by remodeling the gut-skin axis microbiota ecology; whereas at the microscopic level, the epigenetic modification of HDAC3 by Rg3 and the elimination of cancer stem cells by Rh2 demonstrate deep interventional capabilities at the root of diseases. This mechanism-which does not forcibly alter a single indicator but tunes the body's intrinsic endogenous repair capabilities by activating Nrf2, regulating LPA receptors, or restarting autophagy flux-endows ginseng with high robustness in managing chronic, recurrent skin diseases, effectively averting drug resistance and rebound effects, and achieving comprehensive management from "treating existing disease" to "preventing future disease"[33, 93–95, 121–123].

However, despite the ideal mechanistic network has been depicted , the translation into clinical reality faces the dual challenges of "biotransformation variability" and "physical delivery barriers." In the actual physiological environment, the efficient conversion of parent saponins into high-potency effector molecules like CK and Rh1 is often limited by individual differences in gut microbiota. Furthermore, how these components traverse the stratum corneum barrier to precisely reach fibroblasts or immune cells in the dermis remains a significant hurdle. These bottlenecks in pharmacokinetics and bioavailability often determine whether in vitro data can translate into real-world clinical efficacy. Although delivery systems like liposomes and microneedles have shown promise, a significant "technological chasm" remains to be crossed to achieve intelligent, on-demand release and precise targeting [238–240, 242, 248].

Looking to the future, breakthroughs in ginseng dermatology will no longer be limited to discovering new compounds, but will lie in embracing complexity and achieving precision. We need to leverage cutting-edge technologies such as spatial transcriptomics, metabolomics, and network pharmacology to dynamically resolve the interaction weights of the modules in Fig. 2 under different skin microenvironments and identify the true master nodes. On this basis, future R&D should be dedicated to developing next-generation ginseng preparations based on biotransformation principles-such as precise fractions directly enriched with CK or Gintonin-and constructing biomimetic smart delivery systems capable of responding to skin pathological characteristics (such as inflammatory pH or enzymatic environments). Ultimately, the value of all this basic research must return to rigorously designed, stratified multi-center clinical studies. Only by constructing a complete chain of evidence-from standardized components to defined mechanistic networks, and finally to confirmed human efficacy-can we truly elevate this ancient Eastern wisdom into a precision therapeutic solution accepted by modern medical logic and capable of tangibly solving global skin health challenges. In navigating these translational challenges, future investigations must transcend the conventional paradigm of targeting isolated cutaneous lesions, redirecting focus toward the distal ameliorative effects of ginseng on psoriasis-associated systemic comorbidities. Concurrently, it remains imperative to bridge the conspicuous evidentiary gaps in dermatological oncology, particularly concerning basal cell carcinoma. This endeavor must be coupled with the proactive integration of next-generation bio-delivery platforms, such as ginseng-derived exosomes. Ultimately, orchestrating these strategic advancements will not only accelerate clinical translational efficiency but also actualize profound, precision-driven interventions for complex dermatological pathologies.

## Data Availability

No datasets were generated or analysed during the current study.

## Abbreviations

AD: Atopic dermatitis
AP-1: Activator protein-1
AQP3: Aquaporin 3
ARE: Antioxidant response element
BCC: Basal cell carcinoma
CAT: Catalase
CCL: Chemokine ligand
CK: Compound K
COX-2: Cyclooxygenase-2
CSCC: Cutaneous squamous cell carcinoma
CSCs: Cancer stem cells
DNCB: Dinitrochlorobenzene
DNFB: Dinitrofluorobenzene
ECM: Extracellular matrix
EGFR: Epidermal growth factor receptor
EMT: Epithelial-mesenchymal transition
ERK: Extracellular signal-regulated kinases
FLG: Filaggrin
GFRS: Ginseng fruit rare saponins
GOPs: Ginseng oligopeptides
GR: Glucocorticoid receptor
GrDENs: Ginseng-derived exosome-like nanoparticles
GSH-Px: Glutathione peroxidase
GSL: Lipidosomes
GSN: Glycerosomes
HaCaT: Human keratinocyte cell line
HB-EGF: Heparin-binding EGF-like growth factor
HDAC3: Histone deacetylase 3
HDFs: Human dermal fibroblasts
HFF-1: Human foreskin fibroblasts
HH: Hedgehog
HO-1: Heme oxygenase-1
IFN-γ: Interferon-gamma
IgE: Immunoglobulin E
IL: Interleukin
IL-1R: Interleukin-1 receptor
IMQ: Imiquimod
IRAK-1: Interleukin-1 receptor-associated kinase-1
Keap1: Kelch-like ECH-associated protein 1
KRG: Korean Red Ginseng
KRGM: Korean Red Ginseng marc
LPA: Lysophosphatidic acid
LPC: Lysophosphatidylcholine
LPS: Lipopolysaccharide
MAPK: Mitogen-activated protein kinase
MDA: Malondialdehyde
MITF: Microphthalmia-associated transcription factor
MMP: Matrix metalloproteinase
NHEKs: Normal human epidermal keratinocytes
NHDFs: Normal human dermal fibroblasts
NLRP3: NLR family pyrin domain containing 3
NOX4: NADPH oxidase 4
Nrf2: Nuclear factor erythroid 2-related factor 2
PAM: Phenolic acid mixture
PI3K: Phosphoinositide 3-kinase
PPD: Protopanaxadiol
PPT: Protopanaxatriol
PTS: Protopanaxatriol saponins
RG-I: Rhamnogalacturonan-I
ROS: Reactive oxygen species
SASP: Senescence-associated secretory phenotype
SC: Stratum corneum
SCFAs: Short-chain fatty acids
Sirt3: Sirtuin 3
SOD: Superoxide dismutase
SYR: Syringaresinol
TEWL: Transepidermal water loss
TGF-β: Transforming growth factor beta
TPA: 12-O-tetradecanoylphorbol-13-acetate
TRP: Tyrosinase-related protein
TSLP: Thymic stromal lymphopoietin
TNF-α: Tumor necrosis factor-alpha
UV: Ultraviolet
UVA: Ultraviolet A
UVB: Ultraviolet B
VEGF: Vascular endothelial growth factor
WGPA-1-RG: Water-soluble ginseng polysaccharide fraction-1 rhamnogalacturonan-I
ZO-1: Zonula occludens-1
